# Comparative genomics reveals insights into the potential of *Lysinibacillus irui* as a plant growth promoter

**DOI:** 10.1007/s00253-024-13210-6

**Published:** 2024-06-11

**Authors:** Sandra Hilário, Micael F. M. Gonçalves, Inês Matos, Luis F. Rangel, José A. Sousa, Maria J. Santos, Camilo Ayra-Pardo

**Affiliations:** 1 Interdisciplinary Centre of Marine and Environmental Research, CIIMAR, Terminal de Cruzeiros do Porto de Leixões, Av. General Norton de Matos s/n, 4450-208 Porto, Portugal; 2 Department of Biology, Centre for Environmental and Marine Studies (CESAM), University of Aveiro, Campus Universitário de Santiago, 3810-193 Aveiro, Portugal; 3 Biology Department, Faculty of Sciences, University of Porto, Rua do Campo Alegre, s/n, FC4, 4169-007 Porto, Portugal; 4 Present Address: GreenUPorto, Sustainable Agrifood Production Research Centre/Inov4Agro, DGAOT, Faculty of Sciences, University of Porto, Campus de Vairão, 747, 4485-646 Vila do Conde, Portugal

**Keywords:** Bacteria, Biocontrol agents, Biofertilizer, Endophytes, *Phoenix canariensis*, Whole-genome sequencing

## Abstract

**Abstract:**

Members of the genus *Lysinibacillus* attract attention for their mosquitocidal, bioremediation, and plant growth-promoting abilities. Despite this interest, comprehensive studies focusing on genomic traits governing plant growth and stress resilience in this genus using whole-genome sequencing are still scarce. Therefore, we sequenced and compared the genomes of three endophytic *Lysinibacillus irui* strains isolated from Canary Island date palms with the ex-type strain IRB4-01. Overall, the genomes of these strains consist of a circular chromosome with an average size of 4.6 Mb and a GC content of 37.2%. Comparative analysis identified conserved gene clusters within the core genome involved in iron acquisition, phosphate solubilization, indole-3-acetic acid biosynthesis, and volatile compounds. In addition, genome analysis revealed the presence of genes encoding carbohydrate-active enzymes, and proteins that confer resistance to oxidative, osmotic, and salinity stresses. Furthermore, pathways of putative novel bacteriocins were identified in all genomes. This illustrates possible common plant growth-promoting traits shared among all strains of *L. irui*. Our findings highlight a rich repertoire of genes associated with plant lifestyles, suggesting significant potential for developing inoculants to enhance plant growth and resilience. This study is the first to provide insights into the overall genomic signatures and mechanisms of plant growth promotion and biocontrol in the genus *Lysinibacillus*.

**Key points:**

• *Pioneer study in elucidating plant growth promoting in L. irui through comparative genomics.*

• *Genome mining identified biosynthetic pathways of putative novel bacteriocins.*

• *Future research directions to develop L. irui-based biofertilizers for sustainable agriculture.*

**Supplementary Information:**

The online version contains supplementary material available at 10.1007/s00253-024-13210-6.

## Introduction

Chemical fertilizers are frequently used in agriculture to boost crop yields and address the increasing food demand of the growing population (Ahsan and Shimizu 2021). Unfortunately, the overuse of agrochemicals has contributed to several environmental problems, including eutrophication of water bodies, degradation of soil health, loss of biodiversity, and the accumulation of toxic chemicals posing risks to human health (Fenibo et al. 2021). Consequently, the environmental impact of chemical fertilizers has emerged as a significant public concern (Gupta et al. 2015).

The current strategies delineated in the United Nations 2030 Agenda focus on attaining sustainable agriculture, and the European Union’s “Farm to Fork Strategy,” embedded in the European Green Deal, seeks to halve pesticide usage by 2030 (Helepciuc and Todor 2022). Consequently, it is imperative to explore alternative methodologies that prioritize environmentally sustainable agriculture aiming to reduce reliance on chemical fertilizers. Organic inputs, encompassing biofertilizers, biopesticides, slow-release fertilizers, and nanofertilizers are emerging as highly promising strategies for enhancing crop productivity (Kumar et al. 2022). These strategies leverage microorganisms and naturally derived metabolites (de Silva et al. 2019; Hamid et al. 2021). A biofertilizer, defined as a product containing living microorganisms and their metabolic activities, aims to improve soil properties, increase plant nutrient availability, and bolster resistance to biotic/abiotic stress (Du Jardin 2015; Fuentes-Ramirez and Caballero-Mellado 2005). The quest for novel biofertilizers as substitute for chemical fertilizers is driven by their attributes, such as low toxicity, eco-friendliness, biodegradability, and minimal post-harvest contamination (Adhikari et al. 2021).

Plant growth-promoting bacteria living in/on the soil, rhizosphere, and plant tissue as endophytes, such as *Azospirillum*, *Azotobacter*, *Bacillus*, *Mycorrhiza*, *Paenibacillus*, *Pseudomonas*, and *Rhizobium* species, are widely embraced as global biofertilizers (Ahsan and Shimizu 2021; Bhat et al. 2022; Cassán et al. 2020). For instance, while *Azotobacter* and *Rhizobium* are nitrogen-fixing bacteria, *Bacillus* stands out as one of the extensively studied bacteria with a proven ability to promote plant growth (Pal et al. 2015; Tiwari et al. 2019). The genus *Bacillus* encompasses 109 species with validly published names as per the List of Prokaryotic names with Standing in Nomenclature (LPSN) (https://lpsn.dsmz.de/; accessed on 12 February 2024) and has long been recognized for its extensive phenotypic diversity (Gupta et al. 2020). Due to its polyphyletic nature, the majority of species have been thoroughly studied and reclassified into other genera (e.g., *Brevibacillus*, *Lysinibacillus*, and *Paenibacillus*) or groups (e.g., the *Bacillus cereus* group, the *Bacillus subtilis* group) to attain phylogenetic and taxonomic coherence (Nguyen et al. 2019).

Initially placed within the genus *Bacillus*, *Lysinibacillus* underwent reclassification as a distinct genus, as proposed by Ahmed et al. in 2007. The genus *Lysinibacillus*, endospore-forming, Gram-positive, and aerobic bacteria, is defined by the type species *Lysinibacillus boronitolerans* and comprises 22 validly published species, according to the LPSN (https://lpsn.dsmz.de/; accessed on February 12, 2024). Particularly noteworthy is *Lysinibacillus sphaericus*, primarily because of its recognized mosquitocidal activity (Berry 2012). However, recent studies have revealed that various *Lysinibacillus* species exhibit plant growth-promoting traits, including auxin production, phosphate solubilization, siderophore production, and nitrogen fixation (Naureen et al. 2017; Sharma and Saharan 2015; Verma et al. 2016). Furthermore, certain *Lysinibacillus* strains have demonstrated the ability to enhance the growth of spinach (Ahsan and Shimizu 2021) and tomato plants (Sahu et al. 2018) in pot trials, and to increase the corn coleoptile length (Pantoja-Guerra et al. 2023).

Various studies have used whole-genome sequencing and comparative analysis to delve into the intricate bacterial mechanisms that contribute to the promotion of plant growth. Examples include investigations on *Bacillus pumilus* strain SF-4 (Iqbal et al. 2021), *Bacillus subtilis* BS87 (Chandra et al. 2021), *Saccharibacillus brassicae* ATSA2 (Jiang et al. 2023), and *Paenibacillus peoriae* ZBSF16 (Yuan et al. 2022). Numerous studies highlight the plant growth-promoting abilities of *Lysinibacillus* (Ahsan and Shimizu 2021; Naureen et al. 2017; Pantoja-Guerra et al. 2023; Sahu et al. 2018). The study by Pantoja-Guerra et al. (2023) primarily focused on exploring the genetic pathways associated with the production of indole-3-acetic acid (IAA) from a genomic perspective. Nevertheless, to date, no other studies have conducted whole-genome sequencing and annotation for profiling plant growth promotion of *Lysinibacillus* species.

In pursuit of microbial taxa with potential agricultural and biotechnological applications, we isolated three endophytic and endospore-forming bacilli strains from Canary Island date palms (*Phoenix canariensis* Chabaud) resembling *Lysinibacillus* spp. colonies. Thus, the aims of this study are twofold: (1) to determine the exact taxonomic position of these strains using phylogenetic analysis and genome data and (2) to gain important insights into the genomic traits of *Lysinibacillus* spp. that promote plant growth through a comprehensive whole genome comparison.

## Material and methods

### Sampling and bacterial isolates

Mature Canary Island date palms (height ≈ 17 m; diameter ≈ 2.8 m) from a coastal environment in an urban setting located in Porto, Portugal (41° 8′ 50.978′′ N, 8° 39′ 42.994′′ W), serve as ornamental plants in a highly touristic area of the city. Considering that the maintenance activities such as pruning are carried out by the local municipality, our sampling focused on pruned leaves. Leaves were collected and subjected to surface sterilization upon arrival at the CIIMAR Laboratory of Animal Parasitology and Pathology at the University of Porto. Briefly, young and healthy leaflets, measuring approximately 4 cm in length, were cut into three shorter segments. These segments underwent a sequential treatment: first, a 15 sec rinse with distilled water, followed by a 15 sec immersion in 70% ethanol, and then a 30 sec exposure to a commercial bleach solution (NaClO < 8%). Subsequently, the leaf segments were subjected to three consecutive rinses with distilled water (10 sec each), after which they were immersed into 20 mL of Ringer’s solution (125 mM NaCl, 1.5 mM CaCl_2_.2H_2_O, 5 mM KCl, 0.8 mM Na_2_HPO_4_, pH 7.4). The samples were then homogenized using a T 10 basic ULTRA-TURRAX® disperser (IKA-Werke, Staufen, Germany) at 10,000 rpm for 5 min or until the solution turned green and the leaf sample was visibly damaged as described by Rashid et al. (2012). The homogenized solution was filtered through a fluted filter paper and 100 μl of the filtrate (undiluted and diluted 1:10) were plated on *Bacillus cereus* selective agar (mannitol egg yolk polymyxin, MYP) (HiMedia, Modautal, Germany) for isolation of *B. cereus* group (e.g., *B. anthracis*, *B. cereus* sensu stricto, *B. thuringiensis*) and other *Bacillus* species. MYP contains mannitol, which is not fermented by some *Bacillus* spp. and egg yolk lecithin, which forms a precipitate around the bacterial colony after cleavage by the bacterial lecithinase. The medium is made selective by the addition of polymyxin B, which inhibits the growth of Gram-negative bacteria. The inoculated MYP plates were incubated at 30 °C for 24 h.

Single colonies were carefully chosen and streaked four to five times on new MYP plates to obtain pure bacterial colonies. Bacterial glycerol stocks were prepared by adding 500 μL of the overnight-grown cultures to 500 μL of 50% glycerol in a 2 mL screw-cap tube, mixing and storing at − 80 °C until use. Pure cultures of strains B2, B3, and B7 were deposited in the Spanish Type Culture Collection CECT and the Leibniz Institute DSMZ.

### DNA extraction and PCR amplification

Bacterial DNA was extracted using the DNeasy Blood & Tissue Kit (Qiagen, Hilden, Germany) with lysozyme pretreatment, following the manufacturer’s instructions tailored for Gram-positive bacteria. The 16S rRNA and *rpoB* genes were selected as molecular barcodes for bacterial identification. Amplification of 16S rRNA was performed using the primer set 27F/1492R (Supplementary Table S1) following the conditions described elsewhere by Fidalgo et al. (2016). Attempts to amplify the *rpoB* gene using the primer set rpoB1206/rpoBR3202 and the PCR program specified by Ki et al. (2009) for *Bacillus* species were unsuccessful. Consequently, a novel reverse primer, rpoB-R (5′ GGTATCATCCGCATCGGTGCAG 3′), was designed based on the alignment of *rpoB* sequences from available *Lysinibacillus* genomes. Then, the primer set rpoB1206/rpoB-R (Supplementary Table S1) was employed to amplify a portion of the *rpoB* gene, with the following PCR conditions: initial denaturation at 95 °C for 3 min, followed by 40 cycles of denaturation at 95 °C for 30 s, annealing at 55 °C for 30 s, and extension at 72 °C for 1.5 min, concluding with a final elongation step at 72 °C for 5 min. The amplification reactions for both genes were carried out in a 25 μL volume, including 1 μL of DNA template, 1 μL of each primer at 0.4 μM (STAB Vida, Lisbon, Portugal), 6.25 μL NZYTaq 2 × green Master Mix (2.5 mM MgCl_2_, 200 μM dNTPs, 0.2 U/μL Taq polymerase) (Nzytech, Lisbon, Portugal), and 15.75 μL of Mili-Q water. The reactions were performed in a VWR^®^ XT^96^ Gradient thermal cycler (VWR, Darmstadt, Germany). Visualization of the amplified products was achieved under UV light (Gel Doc™ XR^+^, BioRad, Hercules, CA, USA) following electrophoresis in 1.5% agarose gels stained with GreenSafe Premium (Nzytech, Lisbon, Portugal). Subsequently, the PCR amplicons were sequenced by STAB Vida (Lisbon, Portugal).

### Phylogenetic analysis

The nucleotide sequences were analyzed with FinchTV v.1.4.0 (Geospiza, Inc. Seattle, Washington, USA; www.geospiza.com/finchtv). The 16S rRNA sequences obtained from Sanger sequencing were compared to the EzTaxon database (Yoon et al. 2017) for 16S rRNA-based identification and selection of the closest relative type species for phylogenetic inferences. Multilocus sequence analysis using housekeeping genes has proven effective in investigating population structures of endospore-forming bacilli species (Logan et al. 2009). Therefore, annotated genomes of closely related representative strains to our isolates (available in the NCBI database) were used to retrieve their respective 16S rRNA and *rpoB* sequences (Supplementary Table S2).

Sequences were aligned using ClustalX v.2.1 software (Larkin et al. 2007) with pairwise alignment setting (gap opening = 10, gap extension = 0.1) and multiple alignment setting (gap opening = 10, gap extension = 0.2, transition weight = 0.5, delay divergent sequences = 25%). Alignments were optimized and manually edited using BioEdit Alignment Editor v.7.0.5. (Hall 1999) and then concatenated using FaBox webserver v.1.61 (https://users-birc.au.dk/palle/php/fabox/) (Villesen 2007). The newly generated 16S rRNA and *rpoB* sequences were deposited in GenBank.

Maximum likelihood (ML) analyses were conducted in MEGA v.11.0 (Tamura et al. 2021) starting from a neighbor-joining tree automatically generated by the software. Nearest-neighbor-interchange (NNI) was used as the heuristic method for tree inference and 1000 bootstrap replicates were performed. MEGA v.11.0 was also used to determine the best nucleotide substitution model for constructing the ML trees. Maximum parsimony analyses were also performed using MEGA v.11.0 (Tamura et al. 2021) using the heuristic search option with 1000 random taxa additions and tree bisection and reconnection (TBR) as the branch-swapping algorithm. All characters were unordered and of equal weight, and alignment gaps were treated as missing data. Phylogenetic trees were edited in Inkscape Vector software v.1.1 (https://www.inkscape.org) (Free Software Foundation, Inc, Boston, MA, USA).

### Genome sequencing and assembly

To comply with the minimal standards for the use of genome data for the taxonomy of prokaryotes (Chun et al. 2018), a genome-based analysis was conducted. The integrity of the isolated bacterial DNA was assessed through electrophoresis on a 0.8% agarose gel, and the quality and purity were determined using a μDrop™ plate (Thermo Scientific™, Waltham, MA, USA) read on the Multiskan™ GO Microplate Spectrophotometer (Thermo Fisher Scientific, Waltham, MA, USA).

The samples were sent to STAB Vida (Lisbon, Portugal), where library construction was performed using the KAPA HyperPrep kit. The generated DNA fragments were sequenced from 40 ng of genomic DNA on the Illumina Novaseq platform, using 150 bp paired-end sequencing reads. After trimming the low-quality reads from output reads, the quality of the raw data was verified using FastQC software (Andrews 2010). The trimmed sequences were assembled using CLC Genomics Workbench v.12.0.3, based on the De Bruijn Graph (DBG) assembly (Hosseini et al. 2021). QUAST v.5.0.2 (Mikheenko et al. 2018) was used to assess the quality of the assembly and ContEst16S was used to check for contamination (Lee et al. 2017).

### Gene prediction, annotation, and functional analysis

Ab initio prediction and structural annotation of genes and components of genomes were assessed using the GeneMarkS-2 algorithm (Lomsadze et al. 2018) implemented in the NCBI Prokaryotic Genome Annotation Pipeline (Tatusova et al. 2016). The predicted coding sequences were functionally annotated against the NCBI’s nonredundant database (http://www.ncbi.nlm.nih.gov/RefSeq/), the Kyoto Encyclopedia of Genes and Genomes (KEGG) (http://www.genome.jp/kegg/) (Kanehisa and Goto 2000; Kanehisa et al. 2023; Kanehisa 2019), and Gene Ontology (GO) (http://www.geneontology.org/) (Ashburner et al. 2000). Protein sequences were classified based on the Evolutionary Genealogy of Genes: Non-supervised Orthologous Groups (EggNOG) (Huerta-Cepas et al. 2019) database. For the analyzes, *E*-value scores below 10^−5^ were considered as significant.

Tandem repeat sequences (TRs) were located across the genome using the software Tandem Repeats Finder (Benson 1999). Structural RNAs (5S, 16S, and 23S rRNAs) and small non-coding RNAs were annotated by searching RFAM (RNA families) models against the query genome with the Infernal’s cmsearch (Nawrocki and Eddy 2013); and tRNAs were predicted using the tRNAscan-SE tool (Lowe and Eddy 1997).

### ANI, TETRA, and DDH analysis

Overall genome-related indices (OGRIs) were analyzed with the genome sequence of *Lysinibacillus irui* ex-type strain IRB4-01, the endophytic strains B2, B3, and B7, and the closely related ex-type strains *Lysinibacillus boronitolerans* JCM 18776, *L. macroides* LMG 18474, and *L. capsici* PB300. The average nucleotide identity (ANI) and the tetra-nucleotide signature correlation index (TETRA) were obtained using the online tool JSpeciesWS (https://jspecies.ribohost.com/jspeciesws/) (Richter et al. 2016). The digital DNA–DNA hybridization (dDDH) indices were determined online (http://ggdc.dsmz.de/distcalc2.php) using the Genome-to-Genome Distance Calculation (GGDC) v.3.0 (Meier-Kolthoff et al. 2013, 2022).

### Whole-genome alignments, pan- and core genomes, and comparative genomics

Global alignment among the genomes of *L. irui* palm-isolated endophytic strains B2, B3, and B7 from Portugal, and *L. irui* type strain IRB4-01 from Nigeria, was carried out using the progressive MAUVE tool of the MAUVE software v.2.4.0 (Darling et al. 2004). The analysis allowed the identification of highly similar genomic regions (colinear blocks), and visually characterized the structural variations in a linear graphical representation. Core- and pangenome analyzes, along with the construction of a circular chromosomal map containing the four genomes, were performed using the EDGAR v.3.2 web interface (https://edgar3.computational.bio.uni-giessen.de/) (Dieckmann et al. 2021), with strain IRB4-01 serving as the reference genome.

Carbohydrate-active enzymes were predicted using the web-based application dbCAN3 (https://bcb.unl.edu/dbCAN2/) with default settings (Zheng et al. 2023). Pathogenicity determinants of strains B2, B3, B7, and IRB4-01 were predicted using the Virulence Factor of Bacterial Pathogen Database (http://www.mgc.ac.cn/VFs/main.htm) (Chen et al. 2005). The genomes were also analyzed by antiSMASH v.7.0 (https://antismash.secondarymetabolites.org) (Blin et al. 2023) and BAGEL v.4.0 (http://bagel4.molgenrug.nl/) (van Heel et al. 2018) to identify biosynthetic gene clusters (BCGs) associated with potential antimicrobial compounds, such as non-ribosomal peptide synthetases (NRPSs) and polyketide synthases (PKSs), and bacteriocins and post-translationally modified peptides (RiPPs), respectively.

Further genomic analyses were performed using the tools provided by the Center for Genomic Epidemiology (CGE) (https://www.genomicepidemiology.org/). PathogenFinder v.1.1 (Cosentino et al. 2013), VirulenceFinder v.2.0 (Joensen et al. 2014; Malberg et al. 2020), and ResFinder v.4.1 (Bortolaia et al. 2020; Camacho et al. 2009) were used to identify genes involved in bacterial pathogenicity toward human hosts, virulence genes, and antibiotic resistance genes, respectively.

## Results

### Phylogenetic reconstruction of palm-isolated endophytic bacteria

Three endophytic bacterial strains, designated as B2, B3, and B7, were isolated from the leaf tissue of Canary Island date palms. These strains were chosen for further genomic analysis based on their (1) lecithinase activity, evident by the formation of a surrounding zone of white precipitate on MYP plates; (2) resistance to polymyxin B; and (3) morphological similarity to *Lysinibacillus* spp., all key selection criteria. Morphologically, colonies ranged from pale-yellow for strains B3 and B7 to yellow orange for strain B2, with a mucous consistency, round (B2, B3) to irregular (B7) shape, shiny surface, flat elevation, and entire margin.

**Fig. 1:**
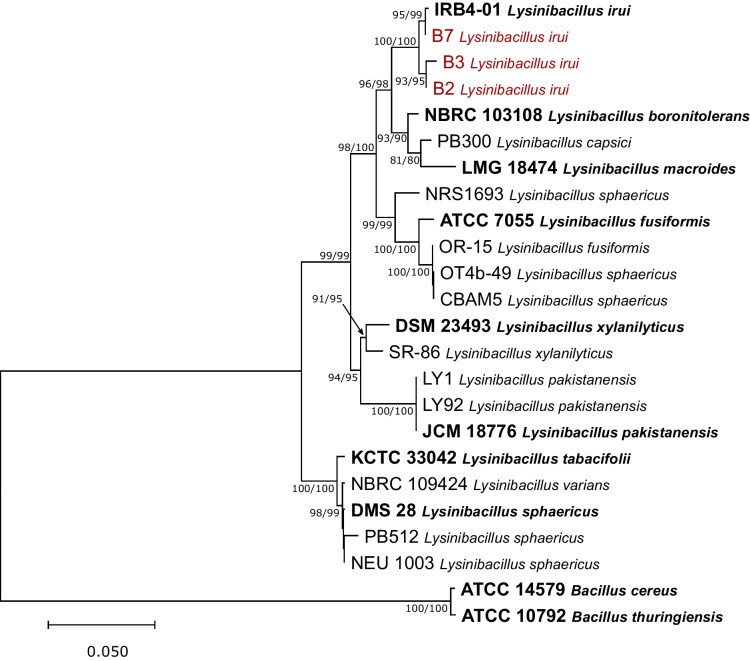
Phylogenetic relationships generated from maximum likelihood analysis based on 16S rRNA and *rpoB* sequence data from *Lysinibacillus* species. The ML tree is rooted to *Bacillus cereus* ATCC 14579 and *B. thuringiensis* ATCC 10792. Maximum likelihood and maximum parsimony (ML/MP) bootstrap values greater than 70% are shown at the nodes. The ex-type strains are shown in bold. The newly generated sequences are shown in red. The scale bar indicates the evolutionary distance

The 16S rRNA and *rpoB* gene sequences of the B2, B3, and B7 strains were obtained through Sanger sequencing and compared with those from whole-genome sequencing. Both sequencing techniques produced identical sequences with a similarity greater than 99.9%. A phylogenetic tree was constructed using multilocus sequence alignments based on the 16S rRNA and *rpoB* genes (Fig. 1). The analysis resulted in the clustering of strains B2, B3, and B7 with *L. irui* ex-type strain IRB4-01, represented by a well-supported clade (MP/PP = 100/100%). Moreover, this clade containing the endophytic strains forms a separate cluster closely related to the type species *L. boronitolerans* JCM 18776, *L. macroides* LMG 18474, and *L. capsici* PB300 (Fig. 1).

### ANI, TETRA, and DDH analysis

The overall genomic relatedness indices were also calculated to investigate the genome similarity between the endophytic strains and the closely related *Lysinibacillus* type species. The results of the genome sequence-based pairwise comparisons are given in Table 1 and show that the threshold of ANI values (95–96%) and TETRA values (> 0.989) for species delineation is surpassed for the endophytic strains B2, B3, B7, and *L. irui* strain IRB4-01. Moreover, the calculation of dDDH by genome BLAST distance phylogeny revealed > 99% genome relatedness of B2, B3, and B7 with IRB4-01, validating their clustering on the same branch of the phylogenetic tree.

**Table 1:** Pairwise comparison of genome sequence-based information of the endophytic strains B2, B3, and B7, and closely related ex-type species of *Lysinibacillus*

Parameters^1^		B2	B3	B7	*L. irui* IRB4-01	*L. boronitolerans* T-10	*L. capsici* PB300	*L. macroides* LMG 18474
B2	ANIb	-	**99.98**	**98.85**	**99.01**	86.45	86.40	82.54
	ANIm	-	**99.98**	**99.21**	**99.25**	87.73	87.66	85.90
	TETRA	-	**1.000**	**0.999**	**0.999**	0.994	0.994	0.970
	dDDh	-	**100.00**	**92.80**	**93.60**	32.20	32.00	27.30
	Prob. ≥ 70%	-	**98.28**	**96.66**	**96.86**	0.25	0.23	0.03
	G + C dif. (%)	-	**0.00**	**0.05**	**0.07**	0.23	0.21	0.55
B3	ANIb	**99.99**	-	**98.84**	**99.02**	86.45	86.41	82.52
	ANIm	**99.99**	-	**99.20**	**99.27**	87.73	87.66	85.90
	TETRA	**1.000**	-	**0.999**	**0.999**	0.994	0.994	0.971
	dDDh	**100.00**	-	**92.78**	**93.60**	32.20	32.00	27.30
	Prob. ≥ 70%	**98.28**	-	**96.69**	**96.85**	0.25	0.23	0.03
	G + C dif. (%)	**0.00**	-	**0.05**	**0.07**	0.23	0.21	0.55
B7	ANIb	**98.77**	**98.77**	-	**99.08**	86.38	86.30	82.70
	ANIm	**99.20**	**99.21**	-	**99.30**	87.72	87.63	85.91
	TETRA	**0.999**	**0.999**	-	**0.999**	0.993	0.993	0.971
	dDDh	**92.80**	**92.80**	-	**94.00**	32.20	32.10	27.40
	Prob. ≥ 70%	**96.66**	**96.65**	-	**96.86**	0.25	0.24	0.03
	G + C dif. (%)	**0.05**	**0.05**	-	**0.02**	0.19	0.17	0.51
*L. irui* IRB4-01	ANIb	**99.12**	**99.12**	**99.13**	-	86.39	86.31	82.59
	ANIm	**99.27**	**99.26**	**99.30**	-	87.72	87.62	85.90
	TETRA	**0.999**	**0.999**	**0.999**	-	0.993	0.992	0.971
	dDDh	**93.60**	**93.60**	**94.00**	-	32.20	32.10	27.40
	Prob. ≥ 70%	**96.86**	**96.85**	**96.86**	-	0.25	0.25	0.03
	G + C dif. (%)	**0.07**	**0.07**	**0.02**	-	0.20	0.20	0.53

^*1*^*ANIb* BLAST-based ANI, *ANIm* MUMmer-based ANI, *TETRA* tetra-nucleotide signature correlation index, *dDDH* digital DNA–DNA hybridization, *Prob* ≥ *70%* probability that the dDDH calculation is 70% or higher, *G* + *C dif.* difference in G + C content (%). Bold values indicate that the strains belong to the same species

### Genomic features of *L. irui* strains B2, B3, and B7

The general features of *L. irui* strains B2, B3, and B7 genomes are summarized in Table 2. From the total genes predicted, the coding regions account for approximately 98.2% of the three whole genomes. Tandem repeats predicted 364, 350, and 391 sequences for strains B2, B3, and B7, respectively, covering 7.7%, 7.4%, and 8.0% of the respective genomes. Among tRNAs, 74 were predicted as anti-codons in all three genomes. Seven ribosomal RNAs, such as 23S (*n* = 1), 5S (*n* = 5), and 16S (*n* = 1), were also found in the genomes. Moreover, ncRNAs including sRNA (*n* = 1), 6S RNA (*n* = 2), ribonuclease P (*n* = 1), and cis-acting RNA elements (riboswitch and ribosomal protein leader regions) were predicted in B2, B3, and B7 genomes.

**Table 2:** Genomic features of *Lysinibacillus irui* strains B2, B3, and B7

Characteristics	Strains		
	B2	B3	B7
Genome size (bp)	4,627,348	4,626,621	4,646,835
GC content (%)	37.2	37.2	37.2
Number of contigs (> 500 bp)	46	38	39
Predicted number of total genes	4734	4738	4867
Average gene length (bp)	840	839	820
Total length of predicted genes (bp)	3,605,388	3,923,619	3,924,402
Predicted no. of coding genes	4648	4652	4781
tRNAs	74	74	74
ncRNAs	5	5	5
rRNAs	7	7	7
Tandem repeat regions	364	350	391
N50 value (bp)	332,082	386,277	260,881
L50 value	5	5	6
Coverage	283 ×	316 ×	239 ×

### Gene prediction and functional annotation

Of the 4734, 4738, and 4867 predicted genes for strains B2, B3, and B7 (Supplementary Tables S3, S4, S5), 14.5%, 14.6%, and 15.6% of the whole genome, respectively, encode hypothetical proteins. Moreover, 69.8%, 82.4%, and 68.4% of genes from strains B2, B3, and B7, respectively, could be annotated with known functions in different COG (Clusters of Orthologous Genes) categories (excluding the poorly characterized category). According to the GO analysis, 47.3% (strain B2), 47.9% (strain B3), and 47.8% (strain B7) of the total predicted genes could also be annotated and assigned to GO terms. Regarding KEGG annotations, 38.9%, 48.4%, and 37.9% of the total genes of B2, B3, and B7 genomes, respectively, were functionally ascribed to KEGG metabolic pathways.

**Fig. 2:**
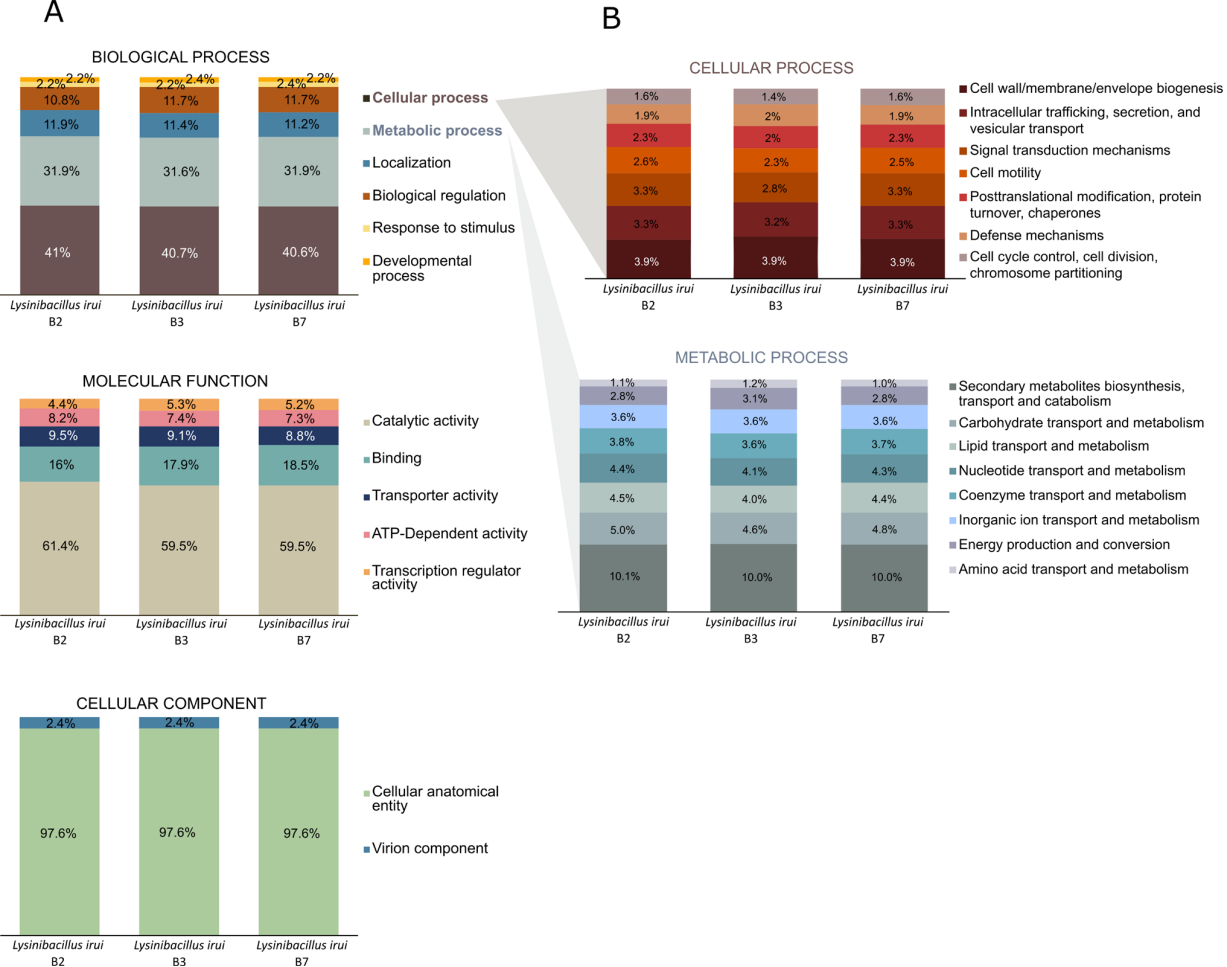
Gene Ontology (**A**) and EggNOG functional annotation (**B**) of *Lysinibacillus irui* strains B2, B3, and B7

Functional analysis of *L. irui* B2, B3, and B7 following the GO annotations revealed that most genes are involved in biological processes including cellular (∼40.8%) and metabolic processes (∼31.8%) (Fig. 2). In the three genomes, genes involved in cellular process category were mostly classified as cell wall biogenesis (∼3.9%); intracellular trafficking, secretion, and vesicular transport (∼3.3%); signal transduction mechanisms (∼3.1%); cell motility (∼2.5%); protein turnover and chaperones (∼2.2%); and others that include defense mechanisms and cell cycle control. In the metabolic process category, the three strains contain genes mainly involved in transport, and catabolism of secondary metabolites (∼10.0%); transport and metabolism of carbohydrates (∼4.7%), lipids (∼4.3%), nucleotides (∼4.3%), coenzymes (∼3.7%), and inorganic ions (∼3.6%); production and conversion of energy (∼2.9%); and transport and metabolism of amino acids (∼1.1%). Regarding molecular functions, genes are mostly involved in catalytic activity (∼60.1%). Within this category, genes are largely ascribed to transferase, hydrolase, and oxidoreductase functions. Genes involved in ion binding processes and transmembrane transporters were also classified within the molecular functions of binding (∼17.4%) and transporter activity (∼9.1%), respectively. Analyses of cellular components showed that most genes encode mostly for cellular anatomical entity functions (∼97.6%) and a few genes in all genomes encode components of bacteriophage virions.

### Comparative analyses

#### Predicted genes and genome statistics

The comparative analysis was performed using the three *L. irui* strains obtained in this study, with the ex-type strain IRB4-01 from Nigeria, to unveil genomic signatures related to plant-growth promotion. Overall, the genomic features of the investigated strains are similar in terms of their GC content and genome size. The GC content of the four strains is approximately 37.3%, the genome size averaged 4.6 Mb per strain, while the predicted coding genes ranged from 4524 to 4781 (Supplementary Table S6). Furthermore, all genomes contained a circular chromosome, and only the genome of strain IRB4-01 was annotated with one plasmid. The analyses performed according to the Center for Genomic Epidemiology (CGE) revealed that all genomes lacked antibiotic resistance genes and genes associated with bacterial pathogenicity in human hosts.

#### Pangenome, core genome, and unique genes

**Fig. 3:**
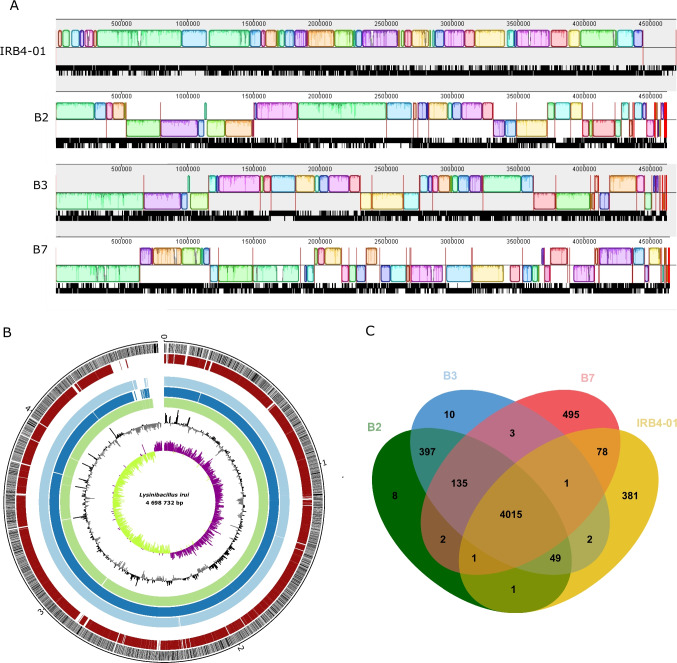
Comparison of *Lysinibacillus irui* genome sequences. **A** Global alignment of *L. irui* genomes B2, B3, B7, and IRB4-01generated using the progressive MAUVE tool. The genome of strain IRB4-01 was used as the reference genome. Red vertical bars represent the start and end of contigs. Boxes with the same color indicate the conserved regions between the genomes. The white regions within the boxes represent sequences with low global similarity, while the white and light gray regions outside the boxes represent unique sequences. Boxes below the horizontal strain line indicate inverted regions. Scale is in nucleotides. **B** Circle genome map. The scale on the outside of the circles indicates the size of *L. irui* genomes; the chromosome is represented by circles ranging from 1 (outer) to 7 (inner). Circle 1: coding sequences (CDS regions) on ± strands; circle 2: *L. irui* strain IRB4-01; circle 3: *L. irui* strain B2; circle 4: *L. irui* strain B3; circle 5: *L. irui* strain B7; circle 6: above and below GC content; circle 7: above and below GC skew. **C** Venn diagram showing the number of clusters of orthologous genes shared and unique genes

Comparative genomic analysis was performed for a global genome collinearity and pan- and core genome analysis. The global genome alignment performed with progressive MAUVE revealed a high similarity between the reference *L. irui* strain IRB4-01 and the endophytic strains B2, B3, and B7 (Fig. 3A), with no large deletions and insertions observed.

The genome collinearity is interspersed with regions of low global similarity (white regions inside the boxes) and unique sequences (white and light gray regions outside the boxes). Moreover, these unique sequences were present in all four genomes and are presented in a circular genome map (Fig. 3B). A total of 5578 genes were identified as the pangenome (Supplementary Table S7), of which 4015 (72% of the total pangenome) were considered core genes (conserved across all genomes) (Fig. 3C) (Supplementary Table S8). The accessory genome (excluding singletons) is represented by 669 genes (12% of the total) (Supplementary Table S9), with individual isolates carrying 103 to 544 accessory genes. The remaining 894 genes are strain-specific, representing 16% of the total pangenome (Supplementary Table S10).

**Fig. 4:**
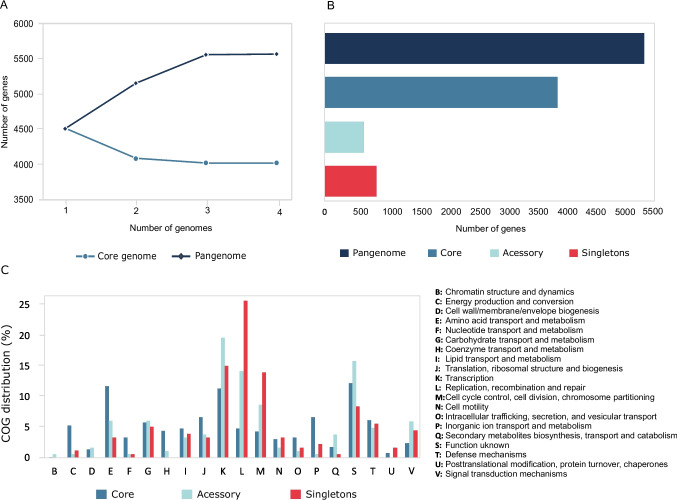
Pan-genome and core-genome profile curves of *Lysinibacillus irui* genomes (**A**). Proportion of genes present in the core, accessory (excluding singletons), and singleton sets for pangenome (**B**). Functional distribution of core, accessory, and singletons of the pangenome of *L. irui* strains according to COG categories (**C**)

The core and pangenome analysis indicated that the inclusion of new genome sequences increased the pangenome size and decreased the core genome size (Fig. 4A). The functional classification of core, accessory, and unique genes (Fig. 4B) according to the EDGAR v.3.2 genome comparison was functionally assigned based on the COG/EggNOG annotation. The COG annotation of the pangenome indicated a great portion of the genome is dedicated mainly to the functional categories: unknown function (12.2%); amino acid transport and metabolism (10.9%); transcription (12.1%); and replication, recombination, and repair (6.6%) (Fig. 4C).

The total of 18,605 protein-coding genes in the genomes of *L. irui* strains B2, B3, B7, and IRB4-01 were grouped into 4015 conserved orthologous genes as shown in a Venn diagram (Fig. 3C). Overall, the core genome consisted of genes related to environmental adaptation, such as *AraC* family transcriptional regulators, DNA repair, and alpha/beta hydrolases. Moreover, virulence factors, CAZymes, flagellar biosynthesis proteins, capsular polysaccharide biosynthesis, sporulation, and chemotaxis proteins were found to be exclusively on the core genome (Supplementary Table S8).

Regarding the strain-specific genes, strains B2 and B3 have the highest number of common genes, represented by 397, but also contain the lowest number of unique singletons. Strain B2 contains eight unique genes, mainly coding for a M15 family metallopeptidase, a bacteriophage holin and DNA polymerase B. Strain B3 is represented by ten unique genes encoding a DNA helicase, zinc-finger-containing proteins, and a phage holin (Supplementary Table S10). Of the 495 unique genes identified in strain B7, 210 genes (50.4%) are annotated as hypothetical proteins, while 174 genes (41.7%) have COG annotations that mainly include function unknown (35.6%); transcription (16.1%); replication, recombination, and repair (15.5%); and cell cycle control (8.0%). The unique genes included, for instance, genes encoding the LexA family transcriptional regulator, bacteriophage/plasmid primase P4 C-terminal domain, siphovirus Gp157 family protein, HNH endonucleases, and the M23 family metallopeptidase (Supplementary Table S10). The genome of the *L. irui* type strain IRB4-01 harbored 381 singletons, of which 223 were annotated as hypothetical proteins (58.5%) and 115 (30.2%) were assigned to the COG categories replication, recombination, and repair (29.0%); function unknown (15.9%); cell cycle control (9.3%); and transcription (6.5%). The unique genes mainly encode IS3, IS6, ISL3 family transposases, type IV secretion systems (T4SSs), Ger(x)C family spore germination protein, AHH domain-containing protein, and the HicB family antitoxin (Supplementary Table S10).

#### CAZymes

**Fig. 5:**
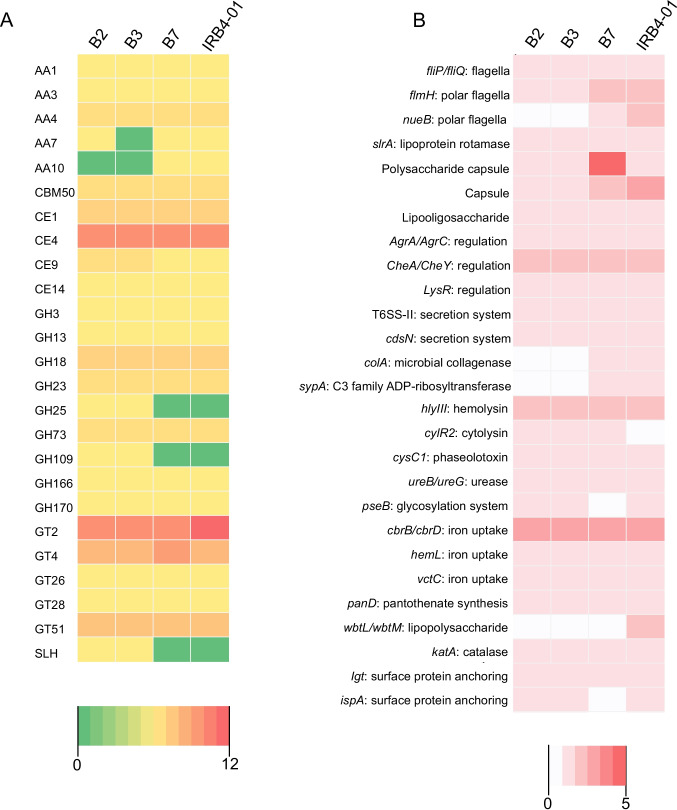
Number of genes predicted in *Lysinibacillus irui* genomes and the respective strains related to carbohydrate-active enzymes (**A**) and virulence factors (**B**)

*Lysinibacillus irui* strains B2, B3, B7, and IRB4-01 display approximately 54 genes encoding putative CAZymes (11 of which carry signal peptides) that were annotated using the HMMER database (Supplementary Table S11). Of these genes, 21 encode glycosyltransferases (GT), 14 encode carbohydrate esterases (CE), an average of 13 genes per species encode glycoside hydrolases (GH), two encode carbohydrate binding modules (CBM), and six encode auxiliary activities (AA), comprising a total of 24 distinct CAZyme families. None of the genomes contained genes coding for pectate lyases (PL) (Fig. 5A). The main GT family includes cellulose/chitin synthases (GT2), β-*N*-acetyl-mannosaminuronyl-transferase (GT26), EPS α-1,6-galactosyltransferase (GT4), digalactosyl-diacyl-glycerol- synthase (GT28), and peptidoglycan glycosyltransferases (GT51) (Fig. 5A). Regarding the CE family, acetyl xylan esterases (CE1, CE4) and *N*-acetylglucosamine 6-phosphate/deacetylase (CE9) were the most abundant.

Carbohydrate binding module 50, a peptidoglycan binding LysM domain, was the only CBM identified in the genomes. Various GH families, including β-glucosidade (GH3), chitinase (GH18, GH23), amylase (GH13), lysozyme (GH25), peptidoglycan hydrolase with endo-β-*N*-acetylglucosaminidase specificity (GH73), α-*N*-acetylgalactosaminidase (GH109), and endo-α-1,4-*N*-acetylgalactosaminidase (GH166) were predicted in the genomes. Notably, GH25 and GH109 families were absent in the genomes of strains B7 and IRB4-01. Within the auxiliary activities (AA) family, multicopper oxidase (AA1), cellobiose dehydrogenase (AA3), and vanillyl-alcohol oxidase (AA4) were found in all genomes. Glucooligosaccharide oxidase (AA7) was not predicted in the genome of strain B3, while lytic polysaccharide monooxygenase (AA10) was absent in B2 and B3 genomes (Fig. 5A).

#### Virulence factors

The Virulence Factor Database predicted virulence factors from different classes in the genomes of *L. irui*, mainly adherence and invasion factors (e.g., flagella biosynthesis), iron uptake factors (e.g., achromobactin biosynthesis and transport), immune invasion (e.g., capsular factors, lipopolysaccharides), regulation factors (e.g., chemotaxis proteins, signal transduction system), secretion systems (e.g., type III and VI secretion system), toxins (e.g., hemolysin III, phaseolotoxin), acid resistance (urease), stress adaptation (catalase), and surface protein anchoring (lipoproteins) (Supplementary Table S12). The genomes of strains B2, B3, and B7 had an annotated toxin virulence factor, cytolysin (*cylR2*), which was absent in strain IRB4-01 (Fig. 5B). Two other annotated toxins were found only in strains B7 and IRB4-01, including a microbial collagenase (*colA*) and the intracellular toxin C3 family ADP-ribosyltransferase (*spyA*). The phytotoxin phaseolotoxin (*cysC1*) was another virulence factor found in all four genomes (Fig. 5B). Apart from members of polysaccharide biosynthesis protein family, which are important factors for biofilm development, only strain B7 contains four additional genes such as an aminotransferase class I/II-fold pyridoxal phosphate-dependent enzyme, a dependent enzyme, a sugar transferase, an UDP-glucose 4-epimerase GalE and a nucleoside-diphosphate sugar epimerase/dehydratase (Supplementary Table S12).

### Genetic elements involved in stress resistance, plant colonization, and plant growth-promoting traits

The predicted proteins of *L. irui* strains B2, B3, B7, and IRB4-01 were searched for their plant growth-promoting-related traits such as biofertilization; phytohormones; root colonization; flagellar motility; and attachment to plant surfaces, stress resilience, volatile organic compounds, and synthesis resistance inducers (Supplementary Table S13).

#### Stress resistance and tolerance

Genome analysis of strains B2, B3, B7, and IRB4-01 revealed the presence of stress tolerance genes including the osmotic stress resistance genes (*opuAC*) and osmoprotectant ABC transporter ATP-binding protein (*osmV*). Several genes important for the regulation of oxidative stress were also detected in the four genomes such as genes for superoxide dismutase enzymes (SOD1, SOD2), peroxide-responsive transcriptional repressor (*perR*), catalase (*katE*), and glutanione peroxidase (*grx*) (Supplementary Table S13). Genes encoding proteins required to neutralize other stressors such as salinity (*argCDEG*) and acidity (sigma-70 family RNA polymerase) were also predicted in the four genomes. We also identified a number of genes related to temperature stress, including a heat-inducible transcriptional repressor (*hrcA*) and a cluster of heat shock proteins (*hrcA-grpE-dnaK-dnaJ*).

#### Spore formation

Several genes regulating sporulation are largely represented in the genomes of the four strains (Supplementary Tables S3, S4, S5). These include the RNA polymerase sporulation sigma factor (*SigG*, *SigK*), outer spore coat protein (*CotE*), sporulation-specific diadenylate cyclase (*CdaS*), and *YlmC*/*YmxH* family sporulation protein and sporulation kinase E (*kinE*). Moreover, spore formation gene clusters are represented in the genomes, such as stage 0 sporulation protein A (*spo0A*), sporulation initiation phosphotransferase (*spo0B*), and sporulation stages I–VII.

#### Flagellar motility, chemotaxis, and root colonization

The genomes of four the *L. irui* strains revealed the presence of several genes encoding flagella basal-body (*flgB*–*flgM*), flagella biosynthesis (*fliO*–*fliR*), flagellin (*fliC*), and motility proteins (*motA*, *motB*). In terms of plant colonization, functional genes for root colonization (dethiobiotin synthase, biotin synthase), and root nodule regulation (glycerol-3-phosphate dehydrogenase/oxidase, glycerol kinase) were also present in the genomes. Moreover, all genomes harbored chemotaxis-related genes (*cheW*, *cheV*) and genes required for bacterial surface attachment. These are involved in both biofilm formation and root colonization, and mainly encode teichoic and lipoteichoic acid (*dltB*C, *dltX*), the carbon storage regulator (*csrA*), and UDP-*N*-acetylglucosamine 2-epimerase (*wecB*).

#### Nitrogen, phosphorus, iron, potassium, and sulfur acquisition

*Lysinibacillus irui* strains B2, B3, B7, and IRB4-01 contain neither a nitrogen fixation cluster (e.g., *nif*) nor genes related to nitrate reductases/nitrite oxidoreductases (e.g., *nar* and *nir*). Regarding phosphate solubilization/acquisition, genes involved in phosphate solubilization, such as glutamate dehydrogenase (*gdh*), aminopeptidase (*iap*), alkaline phosphatase (*phoA*), and the major phosphate-specific transporter system (*pstABCS*) were found on all genomes analyzed (Supplementary Table S13). Genes encoding siderophore production/iron acquisition, such as achromobactin transport system permease (*cbrC*), bacillibactin uptake system (*ybbB*), and iron transport systems involved in the regulation of siderophore uptake (*afuABC*, *fbpABC*, and *feuABC*), were also identified. Potassium-solubilization-related genes including a potassium transporter (*trkG*) and potassium uptake proteins (*trkAH*), and the operon *cys*, which is involved in sulfur metabolism, were also detected in all four *L. irui* genomes.

#### Polyamine production

Genes coding for enzymes involved in polyamine production were found on the four genomes analyzed. These include genes encoding arginine descarboxylase (*speA*), agmatinase (*speB*), spermidine acetyltransferase (*speG*), and polyamine transporter system (*potABCD*) required for putrescine and spermidine biosynthesis.

#### Volatile organic compounds (VOCs)

**Fig. 6:**
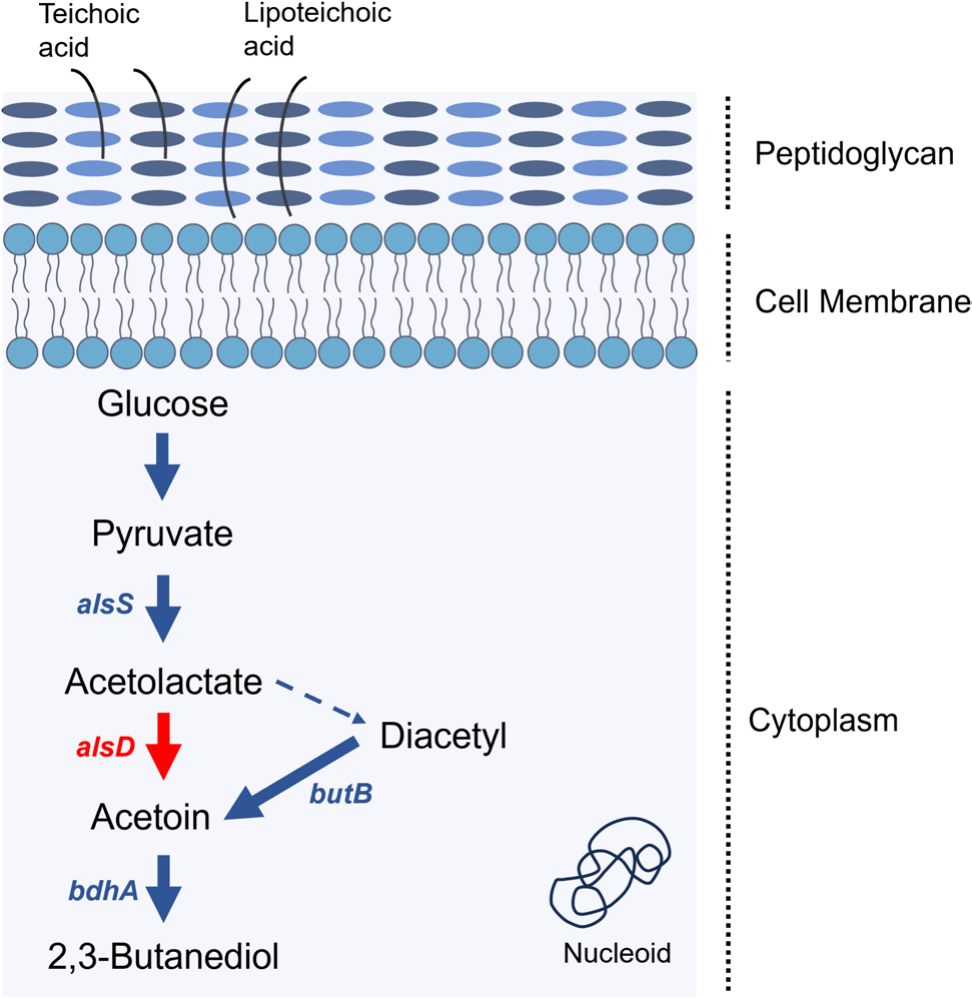
Schematic representation of butanoate biosynthesis pathway in *Lysinibacillus irui*. Blue arrows and blue letters indicate metabolic pathways and genes found in the genomes, respectively. The red arrow and gene name indicate its absence in the genomes. The dotted blue arrow represents the nonenzymatic reaction. *alsS*: acetolactate synthase; *alsD*: acetolactate decarboxylase; *butB*: deacetyl reductase; *bdhA*: butanediol dehydrogenase

The genomes of all *L. irui* strains contain an acetolactate synthase (*alsS*) that converts pyruvate to acetolactate, the first step in the production of acetoin and 2,3-butanediol from the pyruvate pathway. The second step of the pathway is the production of acetoin from acetolactate by an acetolactate decarboxylase (*alsD*), or through the conversion of acetolactate into diacetyl (Fig. 6). Although the *alsD* gene was not present in any of the genomes analyzed, a diacetyl reductase (*butB*) that catalyzes the conversion of diacetyl to acetoin, and a butanediol dehydrogenase (*bdhA*) that catalyzes the conversion of acetoin to 2,3-butanediol, were found in all four genomes.

Moreover, terpenoid VOCs are synthesized by the plastidial methylerythritol phosphate (MEP) pathways in the four *L. irui* genomes. Functional annotation revealed the presence of four genes encoding enzymes involved in isoprenoid precursor biosynthesis (*ispE*, *ispF*, *gcpE*, and *lytB*) (Supplementary Table S13).

#### Pathways of plant hormone production

**Fig. 7:**
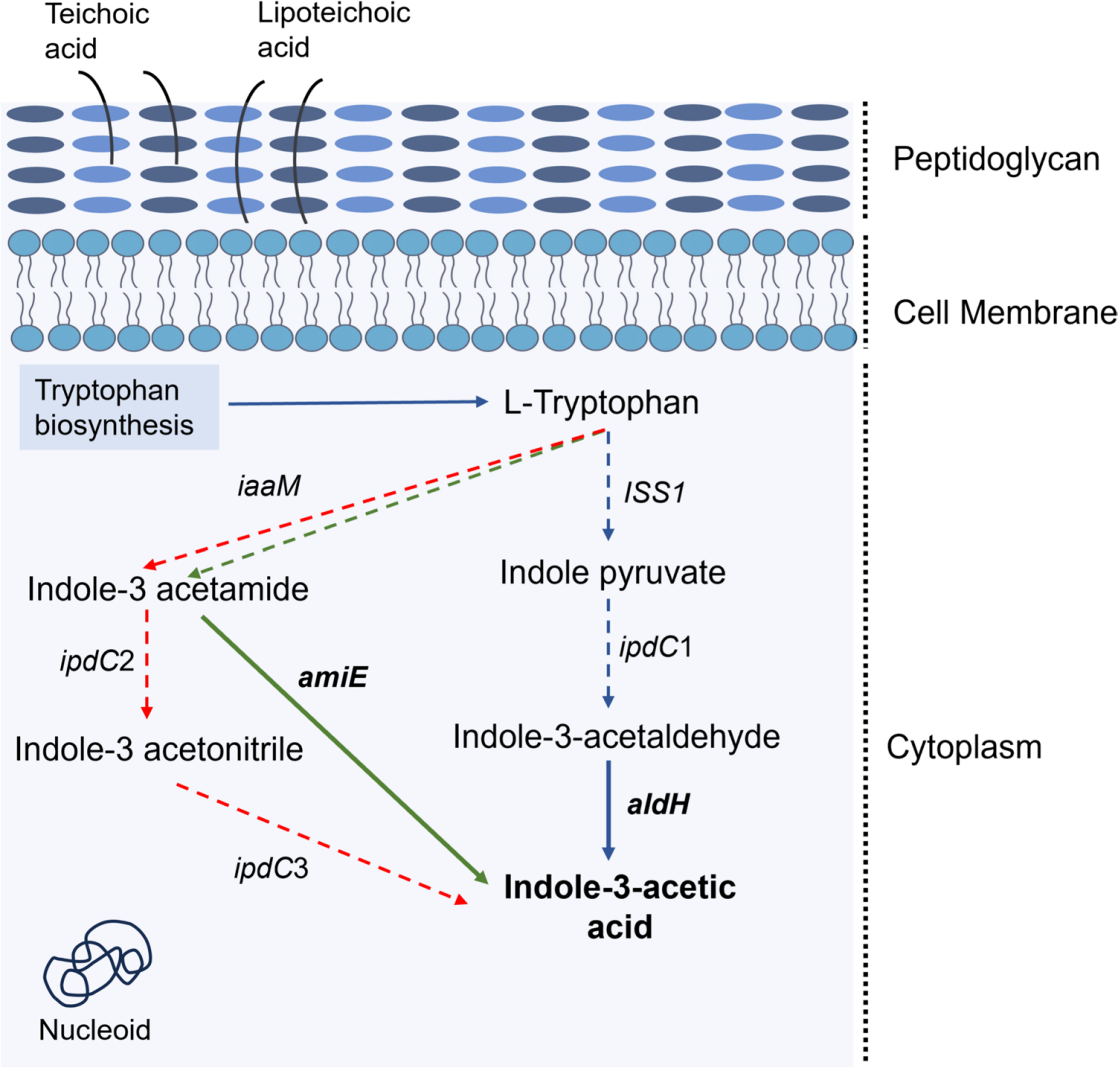
Schematic representation of indole-3-acetic acid biosynthesis pathways in *Lysinibacillus irui*. Blue, green, and red arrows represent the indole-3-pyruvic acid (IPyA), indole-3-acetamide (IAM), and indole-3-acetonitrile (IAN) metabolic pathways. Full arrows and gene names in bold indicate their presence in the genomes. Dashed arrows represent the absence of metabolic pathway in the genomes. *ISS1*, aromatic aminotransferase; *ipdC*1, indolepyruvate descarboxylase; *ipdC*2, nitrile hydratase subunit alpha; *ipdC*3, nitrilase; *aldH*, aldehyde dehydrogenase; *iaaM*, tryptophan 2-monooxygenase; *amiE*, an aliphatic amidase

Regarding plant hormone production, all genomes contain genes encoding enzymes of both the indole-3-acetamide (IAM) and indole-3-pyruvate (IPyA) pathways (Fig. 7), responsible for the production of indole-3-acetic acid (IAA). A key gene is aliphatic amidase (*amiE*), homologous to indole-3-acetamide hydrolase (*iaaH*), which plays a crucial role in the conversion of indole-3-acetamide to IAA. There are also aldehyde dehydrogenases (*aldH*) which convert indole-3-acetaldehyde into IAA and occur in the IPyA pathway. Notably, a gene coding for tryptophan-2-monooxygenase (*iaaM*), which is involved in the conversion of tryptophan to indole-3-acetamide, was not found in the four genomes analyzed.

#### BGCs and bacteriocin-related genes

In *L. irui* genomes under analysis, only three biosynthetic gene clusters (BGCs) were identified, encoding a type 3 polyketide synthase (t3PK), a betalactone and a nonribosomal peptide synthetase-like (NRPS-like) (Supplementary Table S14). Notably, none of these identified secondary metabolite gene clusters exhibited 100% similarity with known BGCs. Some clusters exhibited a limited homology with known secondary metabolites, including the siderophore bacillibactin (30%), the antifungal lipopeptide fengycin (46%), and the antibacterial kijanimicin (4%). This suggests the possibility of gene truncation. For instance, the cluster showing 30% similarity with bacillibactin contains only three genes encoding an ABC transporter substrate-binding protein and two iron ABC transporter permeases. Moreover, the fengycin BGC typically consists of 15 genes, but in *L. irui* strains, it showed 46% similarity and lacked the fengycin synthetases *FenABCD*. Instead, it contained an acetyl-CoA carboxylase (*yngE*), an enoyl-CoA hydratase (*yngF*), a 2-isopropylmalate synthase (*yngG*), a hypothetical protein, an acetyl-CoA carboxylase biotin carboxylase (*yngH*), an AMP-dependent synthetase and ligase (*yngI*), and an acyl-CoA dehydrogenase (*yngJ*).

**Fig. 8:**
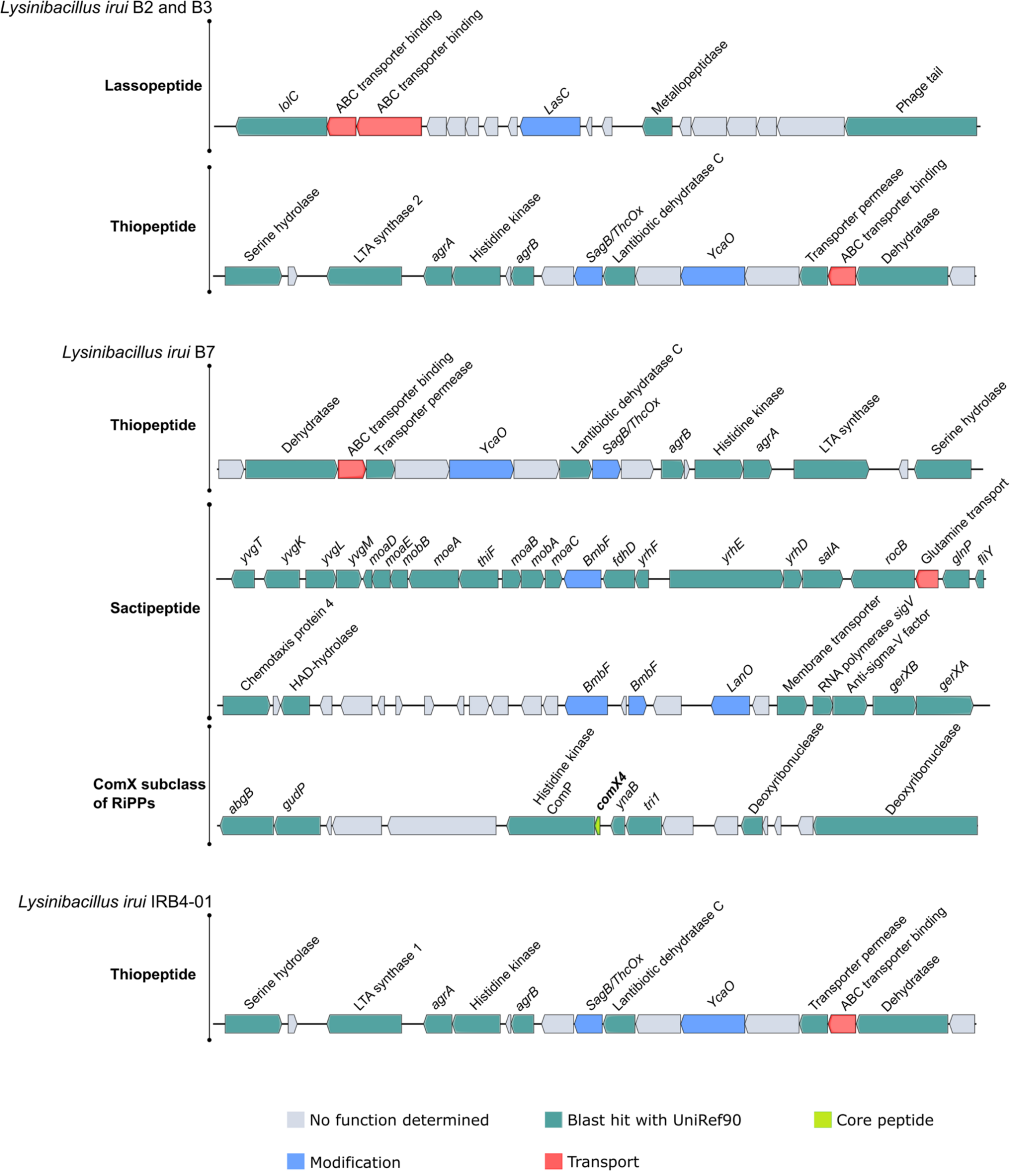
Putative novel bacteriocins identified from *Lysinibacillus irui* strains B2, B3, B7, and IRB4-01. The core peptide is highlighted in bold

The BAGEL analysis yielded one, two, and four areas of interest (AOI) for strains IRB4-01, B2/B3, and B7, respectively (Supplementary Table S15). These regions included genes encoding bacteriocins of the class sactipeptide, lassopeptide, and thiopeptide. From this, one ComX subclass of RiPPs could be classified as a novel bacteriocin, as its core peptide shares less than 70% with known sequences in the BAGEL v.4.0 database (Fig. 8). Apart from the thiopeptide gene cluster, the genome of strain B7 contains a set of genes responsible for producing two putative bacteriocins of the class sactipeptides. Among the 22 genes identified, this cluster includes a *BmbF* gene encoding a GTP 3′,8-cyclase, a glutamine transport ATP-binding protein (*GlnQ*) and permease (*glnP*), crucial for peptide translocation. Furthermore, it involves the molybdenum cofactor biosynthesis operon *moaABCDE*, and the iron-sulfur carrier protein (*salA*) (Supplementary Table S15). The second sactipeptide gene cluster is composed of two *BmbF* genes encoding radical SAM cysteine-rich motif proteins (*YfkA*, *YfkB*), a lanthipeptide oxidoreductase (*LanO*), two spore germination protein (*gerXA*, *gerBX*), methyl-accepting chemotaxis protein 4 (*mcp4*), a sigma-70 family RNA polymerase (*sigV*), an anti-sigma-V factor (*RsiV*), and a 4-toluene sulfonate uptake permease (TSUP family). The RiPPs cluster found in the genome of strain B7 is composed of 16 genes, sharing 39.1% of similarity with ComX4 from the *B. subtilis* group. In particular, ComX4 belongs to the ComX subclass of RiPPs according to the BAGEL4 database and is part of a major quorum-sensing system.

## Discussion

In this study, a whole-genome comparison perspective was implemented to unveil genomic traits related to plant growth promotion in three strains (B2, B3, and B7) of *L. irui* isolated from *P. canariensis* in Portugal and the ex-type strain of *L. irui*, initially isolated from fermented African locust beans, in Nigeria (Akintayo et al. 2023). Our phylogenetic analysis indicated a close relationship between B2, B3 and B7 strains and the *L. irui* ex-type strain, which was further confirmed by ANI and DDH values. Comparative genomics analysis among these four strains revealed an estimated genome size of 4.6 Mb (average per species) which is in line with other *Lysinibacillus* species (Ahmed et al. 2007; Ahsan and Shimizu 2021; Burkett-Cadena et al. 2019). From our analysis, we demonstrated that *L. irui* has an open genome since the pan/core genome profiles tend to increase and decrease, respectively, with the addition of new genomes. Similar results have been noted in previous genomic studies of related *Bacillus* species, including *B. subtillis* (Brito et al. 2018) and *B. velezensis* (Kadiri et al. 2023).

Our study revealed a considerable number of genes encoding carbohydrate-active enzymes (CAZymes) are present in the genome of *L. irui*, on average 54 per species, which enhance the ability of this species to colonize plants (Bhattacharyya et al. 2017). Among these, glycosyltransferases (GTs) and glycoside hydrolases (GHs) were the most abundant enzyme families. While bacteria use GHs to degrade plant polysaccharides and obtain carbon sources for their growth (Johnson et al. 2012), GTs are used to synthesize extracellular polysaccharides for biofilm formation, and thereby to enhance resistance to environmental pressures (Mohnike et al. 2021). Apart from genes for GHs and GTs, all four genomes contained genes for carbohydrate esterases (CEs), with CE4 genes being predominant. CE4 is involved in the deacetylation of polysaccharides, such as xylan, chitin, and peptidoglycan (Oberbarnscheidt et al. 2007). Specifically, enzymes acting in the deacetylation of chitin may be involved in the degradation of fungal or insect cell walls. Therefore, we hypothesize that *L. irui* may be able to colonize plants, degrade plant-derived carbohydrates, as well as to potentially produce enzymes that could be beneficial for the development of biocontrol strategies.

Among the virulence factors detected, the gene *hlyIII*, encoding hemolysin III, was consistently found in all genomes. A recent study revealed that a hydrothermal strain of *B. subtilis* containing the gene *hlyIII* caused lysis of rabbit blood and mortality in fish and mice (Gu et al. 2019). While the role of *hlyIII* in the virulence of *Bacillus* is not yet fully understood (Ramarao and Sanchis 2013), it is crucial to recognize its potential toxicity or lethality to vertebrate animals upon the introduction of *Bacillus*-related species into the environment. Moreover, the four genomes contain the virulence factor phaseolotoxin (*cysC1*). Originally identified in *Pseudomonas* spp., phaseolotoxin induces chlorosis in plants (Bender 1999). However, the phaseolotoxin biosynthesis gene cluster consists of 23 genes (Aguilera et al. 2007; Arrebola et al. 2011). Therefore, further experimentation is necessary to confirm the function of the CycC1 protein in these strains and its potential toxic effects on plants. Furthermore, the gene *colA*, encoding a microbial collagenase of the phospholipase C regulator (*PlcR*) virulence regulon, was found on strains B7 and IRB4-01. Bacterial collagenases, such as those produced by *B. thuringiensis* strains, have been found to exert toxic effects on insects and the soil nematode *Caenorhabditis elegans* (Peng et al. 2016; Wan et al. 2019). Additionally, our genomic analysis identified two *ssrS* genes encoding 6S ncRNA, previously associated with the regulation of insecticidal crystal formation in *B. thuringiensis* (Li et al. 2020). As such, our findings provide a basis for future studies exploring the potential ability of *L. irui* strains as biocontrol agents.

Members of the genus *Lysinibacillus* produce endospores (Akintayo et al. 2023, Ahsan and Shimizu 2021; Burkett-Cadena et al. 2019). In fact, we found several genes from all sporulation stages (from chromosome replication to endospore maturation) in the four *L. irui* genomes. The production of endospores gives the bacterium a significant advantage in resilience to environmental stresses and facilitates the formulation and storage of commercial bacterial-based products (Petrillo et al. 2021). Genes related to motility, mainly flagellar assembly, were also found in the four genomes of *L. irui*. Previous studies have shown that flagella-mediated motility is important for the bacteria to move to favorable environments and enhance bacteria-host interactions (Gu et al. 2019). Moreover, our results revealed that *L. irui* contains polar and lateral flagella, suggesting that these strains can swim and swarm to escape unfavorable conditions and move into plant tissues in search of nutrients. In addition, the presence of genes involved in capsular polysaccharide (CAP) biosynthesis may enable *L. irui* to produce exopolysaccharides, which help the bacteria to colonize plants and protect the host from pathogens (Ramakrishna et al. 2019).

A greater number of function-related genes can help bacteria adapt to complex environments, leading to enhanced adaptability, survival, and growth (Wang et al. 2023). Previous studies have shown that core genes play an important role in controlling essential functions for basic bacterial lifestyle (Yang et al. 2019; Yin et al. 2022). Genomic analysis of *L. irui* strains revealed an abundance of core genes associated with DNA repair and the *AraC* family of transcriptional regulators. Therefore, we hypothesize that the abovementioned genes may play a crucial role in the modulation and control of central carbon metabolism and stress response (Santos et al. 2009; Wang et al. 2023). This, in turn, enables the bacteria to survive under severe environmental conditions. Moreover, all four genomes analyzed contained cytochrome P450 genes, suggesting the ability to enhance the adaptability of plants to the environment by suppressing leaf senescence and extending leaf lifespan (Cowan et al. 2022; Jiang et al. 2021).

Helix-turn-helix domain-encoding genes were identified in all genomes analyzed. This domain is often found in proteins that bind DNA, such as transcription factors. Strains of *L. irui* were also found to harbor genes of the *TetR*/*AcrR* family of transcriptional regulators, which monitor the dynamics of the cellular environment and regulate genes involved in antibiotic production, resistance to osmotic stress, and modulation of cellular metabolism (Deng et al. 2013; Ramos et al. 2005). Other *L. irui* genes encode ABC transporter proteins, alpha/beta hydrolases, and *ArsR* metalloregulators. Thus, this may indicate the potential ability of these strains to sequester and degrade pollutants to improve the adaptability of the bacteria to the environment and thereby mitigate stress conditions caused by such pollutants in the host plants (Santos et al. 2009; Talwar et al. 2020). Nevertheless, to comprehensively ascertain the pollutant-degrading potential of *L. irui*, additional studies are warranted, such as transcriptomics or RT-qPCR analysis in the presence/absence of pollutants.

As mentioned above, *Lysinibacillus* species are reported to harbor plant growth-promoting features such as siderophore production, nitrogen fixation, and phosphate solubilization (Ahsan and Shimizu 2021). Our findings showed that *L. irui* strains contain a glutamate dehydrogenase (*gdhA*) that helps the bacteria to solubilize immobilized phosphate (Sashidhar and Podile 2010). The four *pst* genes (*pstS*, *pstC*, *pstA*, and *pstB*) from the phosphate-specific transport (Pst), an important bacterial transport system for phosphate, were also present in all genomes analyzed. Previously, Yuan et al. (2022) and Jiang et al. (2023) had also reported the presence of *pstABCS* in the plant growth-promoting bacteria *Paenibacillus peoriae* and *Saccharibacillus brassicae*, respectively.

Iron is an essential nutrient required for plant growth but is not available to plants due to its low bioavailability in soil (Jiang et al. 2023). The biosynthetic gene cluster *dhbABCDEF* is responsible for the synthesis of the iron-siderophore bacillibactin, and it has been detected in *Bacillus amyloliquefaciens* (Niazi et al. 2014) and *Bacillus velezensis* (Rabbee et al. 2019). In this study, we detected three genes from this cluster encoding three ABC transporters, suggesting that *L. irui* may not be able to produce bacillibactin. Nevertheless, other iron-siderophores involved in siderophore uptake were identified in the genomes, including a ferric uptake regulator (*perR*), and the iron ABC transporters *afuABC* and *fbpABC*. Furthermore, similar to our study, both ABC transporters in *Saccharibacillus brassicae* (Jiang et al. 2023) and *Virgibacillus halodenitrificans* (Sharma et al. 2023) were identified as siderophore transport systems. Therefore, we suggest that *L. irui* may be able to acquire iron and make it available to plants.

Our results show that all genomes of *L. irui* contain genes encoding enzymes involved in the biosynthesis and secretion of the polyamine spermidine. Polyamines are important features in the alleviation of biotic and abiotic stresses and in the regulation of plant growth and development (He et al. 2023). For instance, Chattopadhayay et al. (2002) reported that spermidine prevents the salt-stress induced leakage of electrolytes and amino acids from rice roots and shoots. Moreover, a polyamine-producing *Bacillus megaterium* was shown to reduce Cd accumulation in spinach (He et al. 2023) and lettuce (Han et al. 2020), suggesting the importance of polyamines in mediating tolerance to heavy metal toxicity.

Indole-3-acetic acid (IAA) is a signaling molecule that plays an important role in promoting plant growth (Tang et al. 2023). Various pathways contribute to IAA production from tryptophan (Zhang et al. 2019). In our analysis, we identified genes encoding enzymes from the indole-3-pyruvate and indole-3-acetamide pathways. From the indole-3-pyruvate pathway, we only found the *aldH* gene, which is involved in the final step of the conversion of indole-3-acetaldehyde into IAA. Of the two genes (*ipdC* and *amiE*) from the indole-3-acetamide pathway, responsible for the conversion of indolic compounds to IAA (Duca et al. 2014), only *amiE* was detected. It is noteworthy that the indole-3-acetamide pathway has primarily been characterized in phytopathogenic bacteria as a virulence mechanism, inducing gall tumors on host plants (Kunkel and Harper 2018; Patten et al. 2013). Therefore, further testing, both in vitro and in planta, is essential to ascertain whether *L. irui* can indeed produce IAA, stimulate root growth and elongation, and importantly, ensure its non-pathogenicity to the host plant.

Volatile organic compounds (VOCs) are a group of molecules synthesized by microorganisms (Chandrasekaran et al. 2023), whose effects result in biotic and abiotic stresses tolerance, plant growth, and inhibition of plant pathogens (Tahir et al. 2017). Among the most common VOCs, three are produced via pyruvate during butanediol fermentation: acetoin, diacetyl, and 2,3-butanediol (Silva Dias et al. 2021). Our analyses revealed a complete pathway for the production of acetoin and 2,3-butanediol, through a non-enzymatic reaction. This reaction is associated with the instability under aerobic conditions, i.e., oxygen-limiting conditions (Cruz Ramos et al. 2000; Silva Dias et al. 2021). For example, Ryu et al. (2003) stated that the low O_2_ pressure in the rhizosphere may stimulate the 2,3-butanediol pathway in *Bacillus*. It has also been suggested that acetoin production and its secretion is a mechanism used by *Bacillus* to maintain the cytoplasmic pH (Kitko et al. 2009). Yi et al. (2016) demonstrated that the application of 2,3-butanediol to the roots of peppers induced the secretion of exudates that modulated the soil rhizosphere. In addition, acetoin and 2,3-butanediol from *Bacillus amyloliquefaciens* were found to induce stomatal closure in *Arabidopsis* and tobacco plants (Wu et al. 2018), while acetoin from *Bacillus velezensis* showed antifungal activity on the rice pathogens *Rhizoctonia solani* and *Magnaporthe oryzae* (Lim et al. 2017). Thus, our findings suggest that *L. irui* could be able to produce acetoin and 2,3 butanediol under oxygen deprivation in the rhizosphere. This, in turn, may favor stomatal closure to prevent infection by microorganisms, induce systemic tolerance to environmental stresses, and maintain cellular pH homeostasis under unfavorable conditions.

The biocontrol potential and the ability of bacteria to promote plant growth is mainly attributed to their secondary metabolites (Jamali et al. 2020; Prasad et al. 2023). These compounds released in the soil are mainly involved in the colonization of the roots and the surrounding niche (Petrillo et al. 2021). Our findings revealed the presence of BGCs encoding for polyketides, beta-lactones, and non-ribosomal peptides that showed a similarity < 70% with known BGCs. Thus, we hypothesize that these BGCs detected in the four genomes may be incomplete or the genes may be truncated, leading to the production of novel and/or related compounds or even non-production.

Bacteriocins are a group of antimicrobial peptides produced by bacteria and used against microbial human and animal pathogens, as well as bacterial plant diseases (Ahmad et al. 2017; Rooney et al. 2020). Our results showed the presence of bacteriocin classes such as thiopeptides, lassopeptides, and sactipeptides. The production of sactipeptides has been reported in various *Bacillus* species, including subtilosin A from *B. subtilis* strain 168 (Kawulka et al. 2003), sporulation killing factor (SKF) from various strains of *B. subtilis* (Engelberg-Kulka and Hazan 2003), and thuricinZ/huazacin from *B. thuringiensis* serovar *huazhongensis* (Hudson et al. 2019; Mo et al. 2019). According to our results, the presence of two clusters encoding sactipeptides in the genome of strain B7 suggests that this strain could potentially produce two novel bacteriocins. Moreover, genomic analysis of strains B2 and B3 revealed the presence of a thiopeptide gene cluster involved in the production of a putative lantibiotic. The LapBotD modification protein (*YcaO*) identified in the cluster is known to play a role in both maturation and production of the antibiotic bottromycin (Williams et al. 2020). Therefore, we hypothesize that both strains B2 and B3 may have the ability to produce a bottromycin-like antibiotic.

The biosynthetic machinery required for lasso peptide production includes genes encoding for the lasso peptide precursor (*LasA*), the leader peptidase (*LasB*), the lasso cyclase (*LasC*), and the ABC transporter (*LasD*) (Arnison et al. 2013). Our findings revealed that the lasso peptide cluster of strains IRB4-01, B2, and B3 contains genes with homology to the *LasABCD* operon. Thus, we hypothesize that these three strains may produce a putative novel lasso peptide. Moreover, strain B7 was predicted to produce a ComX subclass of RiPPs, particularly the ComX4 that regulates the production of surfactins and the quorum-sensing response such as biofilm formation and sporulation in *Bacillus* (Caulier et al. 2019; Chen et al. 2020). Despite the presence of bacteriocin biosynthetic pathways, predicting the production of these compounds by *L. irui* based solely on genomic data is arbitrary. To accurately identify the bacteriocins produced and understand whether they exert antimicrobial activities, the isolation and characterization of the peptides should be conducted for further analysis.

In summary, our study represents a groundbreaking exploration into the genomic traits underlying plant growth promotion and biocontrol mechanisms within the genus *Lysinibacillus*. By employing functional annotation and comparing the genomes of four *L. irui* strains, three of them isolated from palm leaflets as part of this study, we uncovered the presence of pivotal genes associated with phosphate solubilization, acetoin and 2,3-butanediol production, exopolysaccharide and flagella biosynthesis, surface attachment/biofilm formation, and indole acetic acid production. These essential genes were consistently identified within the core genome, indicating shared plant growth-promoting traits across all *L. irui* strains. Additionally, genome analysis unveiled central carbohydrate metabolism and amino acid transport, suggesting the potential utilization of root exudates and plant polysaccharides as energy sources. Notably, the four analyzed genomes consistently harbored genes conferring resistance to oxidative stress, heat shock, osmotic and salt tolerance, metal detoxification, and bacteriocins, making *L. irui* a promising candidate for developing commercial formulations with potential applications in enhancing soil microbiome health, promoting plant growth, and improving resilience to environmental stresses. While this study highlights the genetic potential of *L. irui* in plant growth promotion, further in planta assays are necessary to assess the actual impact on plant performance following bacterial inoculation.

## Supplementary Information

Below is the link to the electronic supplementary material.

Supplementary file1 (XLSX 2778 kb)

## Data Availability

All data generated and analyzed in this study are included in the submitted manuscript and its Supplementary Information file. The Whole Genome Shotgun project of *Lysinibacillus irui* strains B2, B3 and B7 have been deposited in GenBank under the accession number JAXLNX000000000, JAXUHK000000000 and JAXQPV000000000, respectively. The newly generated 16S rRNA and *rpoB* sequences from Sanger sequencing were deposited in GenBank under the accession numbers PP264372–PP264374 and PP273547–PP273549, respectively. The strains obtained and used in this study, were deposited in two publicly accessible culture collections, accessioned CECT 30982 and DSM 117046 (strain B2), CECT 31032 and DSM 117618 (strain B3), CECT 31031 and DSM 117619 (strain B7). Should any raw data files be needed in another format, they are available from the corresponding author upon reasonable request.
